# Anticorrosion Activity of Low-Zinc Powder Coating Primers Containing Single-Walled Carbon Nanotubes

**DOI:** 10.3390/ma18194587

**Published:** 2025-10-02

**Authors:** Barbara Pilch-Pitera, Łukasz Florczak, Dominika Czachor-Jadacka, Francesco Bellucco, Elwira Węgrzyniak-Kściuczyk, Katarzyna Daszykowska, Małgorzata Żychowicz

**Affiliations:** 1 Department of Polymers and Biopolymers, Faculty of Chemistry, Rzeszow University of Technology, ul. Powstańców Warszawy 6, 35-959 Rzeszów, Poland; d.czachor94@gmail.com (D.C.-J.); katarzynamaria1996@gmail.com (K.D.); 2 Department of Physical Chemistry, Faculty of Chemistry, Rzeszow University of Technology, ul. Powstańców Warszawy 6, 35-959 Rzeszów, Poland; 3 Pulverit S.p.A. Italy, Via Carlo Reale 15/4, 20157 Milan, Italy; f.bellucco@pulverit.com; 4 Pulverit Polska Sp. z o.o., ul. Strefowa 16, 43-100 Tychy, Poland; e.wgrzyniak@pulverit.com (E.W.-K.); m.zychowicz@pulverit.com (M.Ż.)

**Keywords:** single-walled carbon nanotubes, epoxy resins, powder coatings, anticorrosion properties

## Abstract

In this work, low-zinc epoxy powder coating primers with anticorrosive properties were developed. For this purpose, single-walled carbon nanotubes (SWCNTs) were introduced into powder coatings. The obtained coatings were evaluated by performing the following tests: adhesion to steel, roughness, gloss, color, water contact angle, salt spray, electrochemical impendance spectroscopy (EIS), and transmission scanning microscopy (TEM). The anticorrosion resistance of the powder coating primers obtained depends on the zinc and SWCNT content, as well as the degree of dispersion in the paint. The mechanism of anticorrosion activity was proposed.

## 1. Introduction

Recently, there has been growing interest in environmentally friendly anticorrosive coating primers, which are used to protect iron, steel, and galvanized surfaces against corrosion. Anticorrosive coating systems are applied in a wide range of industries, including household appliances, machinery, tools, the automotive sector, shipbuilding, construction, and the aviation industry [1].

Currently, the most commonly used anticorrosive coatings are high-zinc organic coatings based on epoxy resins, containing zinc dust in the amount of up to 80–90% and organic solvents (VOC) [2]. In addition, the greater effectiveness of protection with high-zinc primers due to low rust creep has led to even greater pressure to maximize the use of zinc dust in anti-corrosion products. In the case of powder coatings, the need for homogenization in the molten state during production limits the introduction of a large amount of zinc due to the very high melt viscosity, which makes it impossible to carry out this process. Additionally, the high zinc content, due to high friction forces, causes rapid wear of equipment in the production and spraying processes. For the above reasons, the zinc content in powder coatings is lower than 60%, most often below 40%.

The anticorrosive performance of zinc-containing coatings is primarily based on cathodic protection. Zinc undergoes oxidation, forming a protective layer that shields the steel substrate from corrosion. As zinc oxidizes, the coating gradually loses its electrical conductivity, leading to a reduction in galvanic protection. At this stage, the coating continues to provide corrosion protection through barrier and inhibitory mechanisms, primarily due to the presence of zinc corrosion products [3]. However, this type of protection may be insufficient in environments with high levels of corrosivity, such as those classified as C5-M.

The classification of zinc as a hazardous substance has contributed to the search for alternative solutions [4]. In many formulations, zinc dust has been used in combination with zinc flakes [5], stainless steel flakes [6] or has been replaced by phosphate-based pigments, predominantly zinc phosphate [7,8]. This applies to most zinc-free commercial anticorrosive products currently available on the market. However, recent studies have demonstrated the harmful effects of phosphates on aquatic organisms, leading to a gradual restriction of their use according to Directive 2000/60/EC [9,10].

Moreover, the zinc phosphate exhibits a weaker anticorrosive performance compared to metallic zinc. Therefore, to ensure adequate corrosion protection, the application of thicker coatings is recommended. However, this results in increased paint consumption and can negatively affect other coating properties, such as adhesion to the substrate, impact resistance, and flexibility—parameters to which powder coatings are particularly sensitive. Although adhesion can be improved through excellent surface preparation, achieving optimal surface conditions can be challenging in certain cases, such as when the object to be protected is exposed to polluted air or has complex geometry.

The literature also reports numerous studies on the modification of high-zinc anticorrosive primers with various substances, including polyaniline [11,12], aluminum phosphate [13], carbon black, polypyrrole [14] and polyindole [15] to reduce the overall zinc content. However, to achieve corrosion resistance at a comparable level, relatively high concentrations of these modifiers are often required, which significantly increases formulation costs due to the substantially higher price of these materials compared to zinc powder.

Recent scientific studies have also focused on extending the duration of cathodic protection in coatings through the incorporation of conductive nanoparticles, such as conductive carbon allotropes, including conductive carbon black [16], graphene [17,18,19,20] and carbon nanotubes [21,22,23,24,25,26,27,28]. These novel nanomaterials, when dispersed properly within the polymer matrix, can form an electrically conductive network that enables effective anticorrosive protection even at very low nanoparticle loadings, often below 0.1 wt%. This allows for a reduction in zinc content while maintaining the same level of corrosion protection. The main drawback of these advanced solutions is the high cost of nanoparticles; however, this is offset by the small quantities required to achieve a significant effect. Growing industrial interest in these solutions may contribute to the development of new methods for increasing the scale of production of these nanomaterials, which will be associated with a decrease in their prices in the near future.

The use of multi-walled carbon nanotubes (MWCNTs) as anticorrosive additives has also been described in patent no. P.232158 [29], which concerns the modification of both solvent-based and waterborne coating systems. The method of modifying solvent-based epoxy coatings with MWCNTs has been disclosed in patents WO2015132366A1, EP2931818A1, and CN112143340A as well as described by Cubides et al. [30,31,32,33]. Furthermore, patent CN111662623A describes the use of MWCNTs to enhance the anticorrosive performance of high-solid coatings based on polyaspartic resins and polyisocyanates, while patents CN107573818A and CN109971313A relate to their application in epoxy resin-based formulations [34,35,36].

Li et al. used TiO_2_-MWCNTs nanohybrid material on the corrosion resistance of epoxy low-zinc coatings [37].

There are significantly fewer reports in the literature concerning the modification of anticorrosive coatings using single-walled carbon nanotubes (SWCNTs). SWCNTs have been incorporated into corrosion inhibitory nanocomposite solvent-based coatings, such as PMMA/siloxane–silica systems [38,39] and poly[(3,5-dimethyl-1H-pyrazole-1-yl) methyl methacrylate-co-glycidyl methacrylate] coatings [40]. However, there are no reports in the literature on the modification of zinc-rich epoxy powder coating primers using single-walled carbon nanotubes (SWCNTs).

Considering the beneficial effect of single-walled carbon nanotubes (SWCNTs) on the anticorrosion performance of solvent-based acrylic coatings, this study focuses on the modification of zinc-rich epoxy powder coating primers.

The objective of this work was to investigate the influence of SWCNTs and their dispersion method on the anticorrosive properties of zinc-rich epoxy powder coating primers while simultaneously reducing the zinc dust content. The morphology, physical properties, surface free energy, and electrochemical behavior of the epoxy powder coatings were evaluated in relation to different SWCNT dispersion techniques. Furthermore, the impact of SWCNTs and their dispersion methods on the corrosion resistance of the coatings was assessed, and the underlying anticorrosion mechanisms were proposed.

## 2. Experimental Section

### 2.1. Preparation of Powder Coatings Primer

Raw materials:

- Epoxy resin: Eponac 825, Epoxy type 3 (Sir Industriale, Macherio, Italy)
- DICY-based curing agent: Vicura MC-2844 (1-o-tolylbiguanide) (Vesta Chemicals, Zwolle, The Netherlands)
- degassing agent: Benzoin (Aldrich, Buchs, Switzerland)
- flow control agent: Byk 360P (Byk-Chemie, Wesel, Germany),
- zinc dust: 4P32 (EverZinc, Liège, Belgium),
- barite (natural, grinded mineral)
- graphene single-walled carbon nanotubes (SWCNTs) in polyethylene wax Tuball: outer diameter: 1.6 ± 0.4 nm, length: >5 µm, G/D ratio: >90, specific surface area of 1 g SWCNT: ≥300 m^2^, number of SWCNTs in 1 g: 10^17^ pcs (OCSiAl, Leudelange, Luxembourg).

The qualitative and quantitative compositions of the powder coatings are given in Table 1. In order to manufacture powder primer containing SWCNT, two dispersion methods were used. In the first method, SWCNTs were mixed with all raw materials. In the second method, SWCNTs were first mixed with melted epoxy resin at 110 °C and cooled. Then, the mixture of SWCNT and epoxy resin was added to all raw materials. Both mixtures were ground and extruded in an EP 21-25 (TSA, Luisago, Italy) co-rotating twin-screw extruder with an L/D ratio (screw length/diameter) of 25 and a capacity of 15–40 kg/h, equipped with a TSA DVM21 single-screw feeder with a volume of 5 L. Temperature distribution in the extruder was as follows: 60 °C (zone I), 90 °C (zone II), 110 °C (zone III) and 120 °C (an adapter). The screw’s rotational speed was 150 rpm. After extrusion, the paint extrudates, after cooling, were formed into a ribbon and crushed into “chips” using the RNP 500 two-roll calender (Secchi Giovanni, Cernobbio, Italy) with a roll diameter of 160 mm and a length of 200 mm, and a crushing device. The last stage consisted of grinding chips into a fine powder and sifting the paint through sieves to separate particles that were too large or too fine using an ALPINE Classifier Mill ACM 2 EC (Hosokawa Alpine, Augsburg, Germany) with particle separation and a capacity of 35–70 kg/h. The samples obtained according to the first method were marked at the end with the symbol “I”, while the second method was marked with the symbol “II”.

### 2.2. Application on the Substrate and Cross-Linking of the Coatings

The powder coating samples were applied to the sandblasted steel substrate using PEM X-1 CORONA gun controlled by EPG Sprint X from Wagner (Alstatten, Switzerland) and cured at 180 °C for 15 min.

### 2.3. Measurements

Measurements of the current in the powder cloud were made with a Static Check STC MIKA meter (INTEC Maschinenbau, Dortmund, Germany).

A Mar Surf PSI profilometer was used to measure the roughness of cured powder coatings in accordance with the PN-EN ISO 12085 standard [41].

A micro-TRI-gloss µ tester from BYK-Gardner (Geretsried, Germany) was used to determine the gloss of the cured powder coatings according to PN-EN ISO 2813 [42].

The thickness was measured with micro-TRI-Gloss-µ tester from BYK-Gardner according to PN-EN ISO 2808 [43].

The relative hardness of the coatings was determined using König Pendulum manufactured by BYK-Gardner (Geretsried, Germany), according to PN-EN ISO 1522 [44]. The pendulum of a 200 ± 2 g weight was set in motion, and the time at which the amplitude of the oscillation decreased by a predetermined amount was measured. The test was performed in three locations of the coated steel plates, and two plates of each composition were examined.

The adhesion of the coatings to the steel surface was evaluated using a cross-cut test according to the PN-EN ISO 2409 standard [45].

The measurement of the water contact angle was carried out by the side drop method using an optical goniometer by Data Physic (Filderstadt, Germany), model OCA 15, in accordance with the EN 828:2000 standard [46]. The surface free energy was determined using the Owens-Wendt method for the reference liquid pair water-diiodomethane.

Surface resistivity was measured using a Keithley 6517B electrometer (Tektronix, Beaverton, OR, USA) in accordance with EN ISO 3915 [47]. Sample supply voltage U = 2 V. The values are the arithmetic average of 25 results of post-surface resistivity measurements, each repeated in four axes of sample location relative to the chamber for each of three samples of each type (average of 300 measurements).

Zinc leaching tests from the coatings were conducted by completely immersing each sample in 100.0 cm^3^ of artificial seawater. All samples were characterized by the same surface area, 18.75 cm^2^.

The samples were immersed for 28 days at room temperature. The amount of released Zn was measured after 7, 14 and 20 days using the Flame Atomic Absorption Spectroscopy (FAAS) method. The final result is the sum of the measurements after 7, 14 and 28 days.

FAAS measurements were performed using a Perkin Elmer 3100 atomic absorption spectrometer (Perkin Elmer, Waltham, MA, USA). Zinc determination was carried out under the following conditions: analytical wavelength 214 nm, slit 0.7, sensitivity 0.018 mg/dm^3^, linear range 1.0 mg/dm^3^, standard solution concentrations 1.0, 3.0, 5.0 and 6.0 mg/dm^3^.

The composition of artificial seawater per 1 dm^3^ (based on the composition of natural seawater according to: 27.21 g NaCl, 3.81 g MgCl_2_, 1.66 g MgSO_4_, 1.26 g CaSO_4_, 0.86 g K_2_SO_4_, 0.12 g CaCO_3_, 0.08 g MgBr_2_ [48].

The TestAn NS8000 spectrophotometer (Anticorr, Gdańsk, Poland) was used to obtain the color assay according to the standards PN-ISO 7724-1 and PN-ISO-7724-2 [49]. Color was characterized by CIE coordinates in the color space 1979 (L*, a*, b*). The horizontal axes a* and b* are perpendicular to each other, and their intersection is a point of achromatic color. The vertical L* axis describes the brightness level of the color and ranges from 0 to 100.

The color change ΔE was calculated according to Equation (1):

ΔE* = ((ΔL*)^2^ + (Δa*)^2^ + (Δ*b*)^2^)^1/2^ (1)

TEM analyses were performed using a JEOL JEM2100 HT CRYO LaB6 transmission electron microscope (JEOL, Tokyo, Japan). The coating samples were cross-linked in an oven on Teflon film and then cut into ultra-thin slices using an EM UC7 Leica ultramicrotome (Leica Microsystems, Wetzlar, Germany) with an EM FC7 cryo chamber (Leica Microsystems, Wetzlar, Germany).

A Parstat 2273 electrochemical station (Princeton Applied Research, Houston, TX, USA) was used to measure the open circuit potential (OCP) and electrochemical impedance spectroscopy (EIS) in a 3.5 wt% NaCl solution at ambient temperature. The measurements were carried out in a three-electrode system, where the working electrode (WE) was an isolated circular fragment of the coating, the counter electrode (CE) was a platinum plate, and the reference electrode (RE) was a silver chloride electrode. Finally, all potential values were presented relative to a saturated calomel electrode (SCE). EIS measurements were conducted using an applied AC signal with an amplitude of 10 mV (rms) at the open circuit potential in the frequency range of 100 kHz–10 mHz. All electrochemical experiments were performed in duplicate to ensure reproducibility of the electrochemical behavior. Equivalent circuits were fitted to the electrochemical impedance spectroscopy testing data using the ZSimp Win 3.21 fitting software (EChem Software, Ann Arbor, MI, USA).

The corrosion resistance was also assessed by an immersion test (PN-EN ISO 2812-1 [50]). The samples were placed for 720 h in a 3.5% NaCl aqueous solution. Before the test, X type incisions were made on the coating according to PN-EN ISO 17872 [51]. After the test, the samples have been evaluated using the PN-EN ISO 4628-8 standard [52].

## 3. Results and Discussion

### 3.1. Physical and Mechanical Properties

The uncured epoxy powder coating primers were tested for their sprayability using standard powder coating spray guns. The current intensity in the powder cloud is a typical parameter used to characterize the powder’s ability to become electrostatically charged during application. If a spray current of >1.7 μA is reached, the powder being tested is suitable for Corona or Tribo spraying [53]. The measured current intensity in the powder cloud for the prepared samples is presented in Table 2.

The current intensity in the powder cloud for typical powder coatings without any conductive or anti-corrosive additives is usually in the range of 2–3 µA. The current intensity in the powder cloud for all tested samples was relatively high (in the range of 5.4–8.4 µA), which indicates that the addition of conductive particles (zinc and nanotubes) had a positive effect on increasing the sprayability of these powder coatings. The highest current intensity (8.4 µA) was observed in the EP/30Zn powder coating, which contained 30% zinc. The EP/10Zn/0.5CN/I and EP/10Zn/0.5CN/II showed lower current values of 5.4 µA and 6.3 µA, respectively, as a result of reducing the zinc content to 10%. In these powder coatings, 20% of the zinc dust was replaced with 0.5% SWCNT. However, 0.5% SWCNT has a weaker effect on increasing current intensity than 20% zinc. The current intensity of the EP/10Zn/0.5CN/II sample is higher than that of EP/10Zn/0.5CN/I, which indicates a better charge acceptance capacity of EP/10Zn/0.5CN/II. This is due to the better dispersion of SWCNT in this sample as a result of their prior dispersion with melted epoxy resin, which was confirmed by TEM.

Cross-linked powder coatings without defects, such as orange peel, cratering, or pinholes, were obtained and tested for visual, mechanical, and zinc leaching properties. The measured parameters are also included in Table 2.

The surface roughness of the coatings was evaluated using the average parameters R_a_ and R_z_. The measured roughness values for all samples were characteristic of smooth coatings. However, the roughness of the EP/30Zn coating (*R*_a_ = 0.76 ± 0.02 µm, *R*_z_ = 3.92 ± 0.45 µm) was higher than that of the EP/10Zn/0.5CN/I and EP/10Zn/0.5CN/II coatings. Replacing 20% of zinc dust with 0.5% SWCNTs reduced the surface roughness, indicating improved homogeneity. Furthermore, due to the better dispersion of SWCNTs in the EP/10Zn/0.5CN/II sample, a further decrease in surface roughness was observed compared with EP/10Zn/0.5CN/I, suggesting enhanced integration with the epoxy matrix.

The gloss of a coating depends on its roughness. Coatings with lower roughness had higher gloss values.

The thickness of the tested coatings is within the range 98.7–103.0 µm. These thickness values were similar and were within the typical range for powder coatings.

Replacing 20% of zinc dust with SWCNTs and barite resulted in an increase in relative hardness compared to the EP/30Zn coating. This effect is likely due to the presence of barite, which has a higher Mohs hardness (3–3.5) [54] than zinc (2.5) [55], as well as the inclusion of SWCNTs, which can withstand pressures up to 25 GPa without deformation and subsequently transform into superhard-phase nanotubes [56]. The higher hardness value observed for the EP/10Zn/0.5CN/II coating is attributed to the improved dispersion of SWCNTs in the epoxy matrix.

For all tested coatings, the edges of the cuts were completely smooth, without no detachment of the squares in the cut grid (the coatings obtained the highest adhesion class, i.e., 0). Adhesion to the steel substrate was high due to the presence of polar functional groups, such as secondary hydroxyls, formed during the epoxy curing process with the DICY-based curing agent. These hydroxyl groups engage in electrostatic interactions with the steel surface, thereby enhancing adhesion.

The water contact angle is a measure of a coating’s hydrophobicity (water resistance). For hydrophobic coatings, the water contact angle exceeds 90°. Based on the conducted measurements, all tested coatings exhibited hydrophobic character, as their water contact angles were greater than 90°. However, in the SWCNT-containing samples, a decrease in water contact angle was observed, despite the inherently hydrophobic nature of SWCNTs. This effect can be attributed to the presence of 19.5% barite in these formulations, which imparts hydrophilic properties due to its hydrophilic character. Higher contact angle values with the apolar liquid diiodomethane in the SWCNT-containing samples indicate an increase in oleophobicity. A slight increase in water and diiodomethane contact angles observed for the EP/10Zn/0.5CN/II sample is the result of better SWCNT dispersion. The increase in diiodomethane contact angle corresponded to a decrease in surface free energy.

The EP/30Zn sample exhibited the highest surface resistivity (1.2 × 10^12^ ± 0.20 × 10^12^ Ω), indicating poor electrical conductivity, as the surface resistivity exceeds 10^13^ Ω [57]. The samples containing SWCNTs were found to have lower surface resistivity values, indicating improved electrical conductivity. The EP/10Zn/0.5CN/II sample exhibited significantly lower surface resistivity (12.6 × 10^3^ ± 0.08 × 10^3^ Ω) than EP/10Zn/0.5CN/I (1.3 × 10^9^ ± 0.20 × 10^9^ Ω), due to better dispersion of SWCNTs, which came into contact with each other and formed an electrically conductive network. The surface resistivity of this sample is below 10^6^ Ω, which qualifies it as a material with electrostatically conductive properties. This means that it is not capable of accumulating electrostatic charge on its surface, which is highly advantageous for applications in explosive or hazardous environments [58].

The amount of zinc leached into seawater from the tested coatings decreased with the reduction in the zinc content in the formulation. Moreover, this value was not affected by the SWCNT dispersion method.

All coatings exhibited a grey color with a green-blue tinge. The color difference between the reference sample and EP/10Zn/0.5CN/I was less than 2, which is not perceptible to the average observer. In contrast, the color difference between the reference sample and EP/10Zn/0.5CN/II was ΔE* = 3.48. The largest contribution to the color difference came from the L* component, indicating that the sample had a darker shade. This effect results from a better dispersion of the SWCNTs, which are inherently black in color.

### 3.2. Morphology

**Figure 1:**
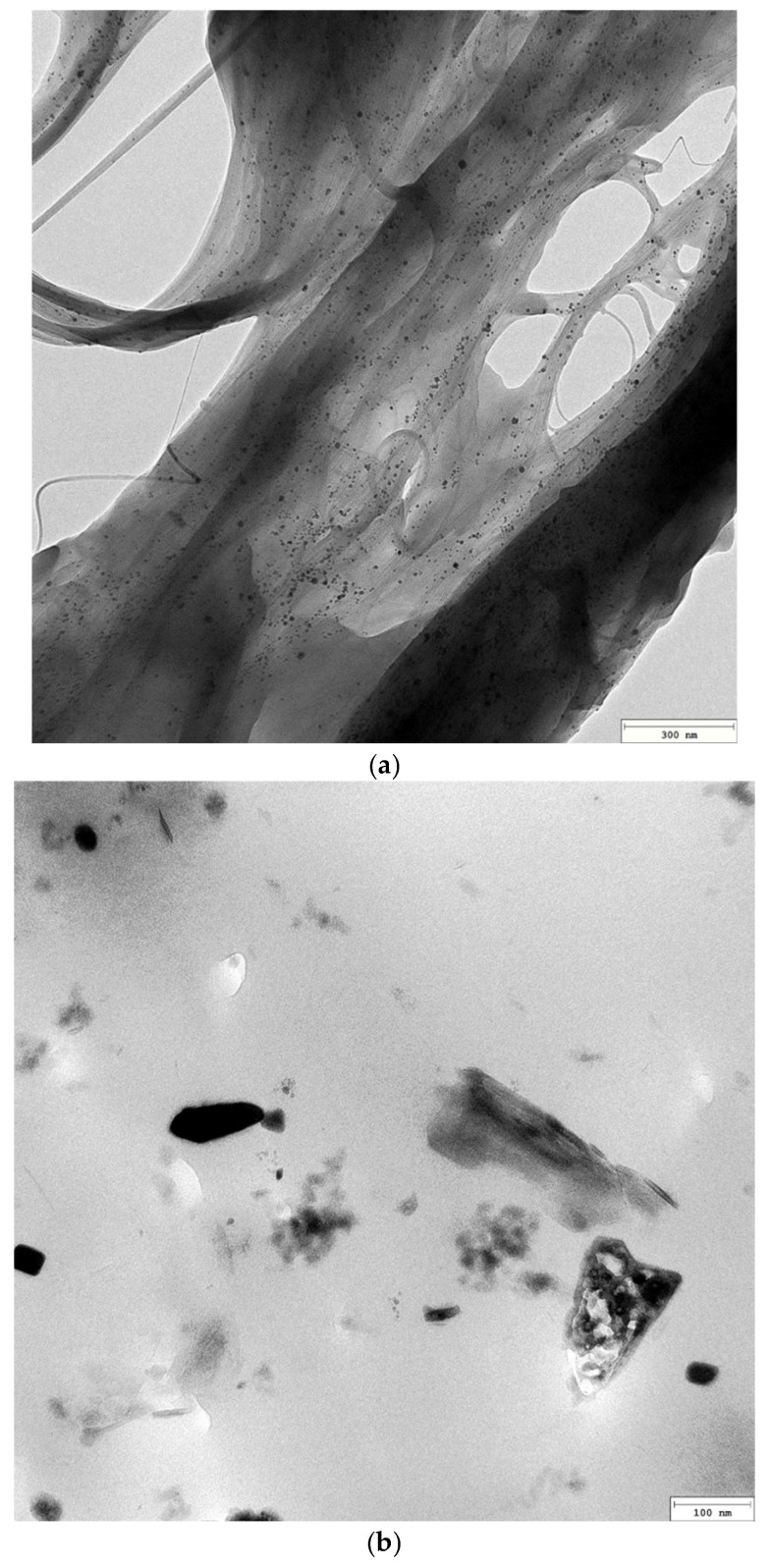

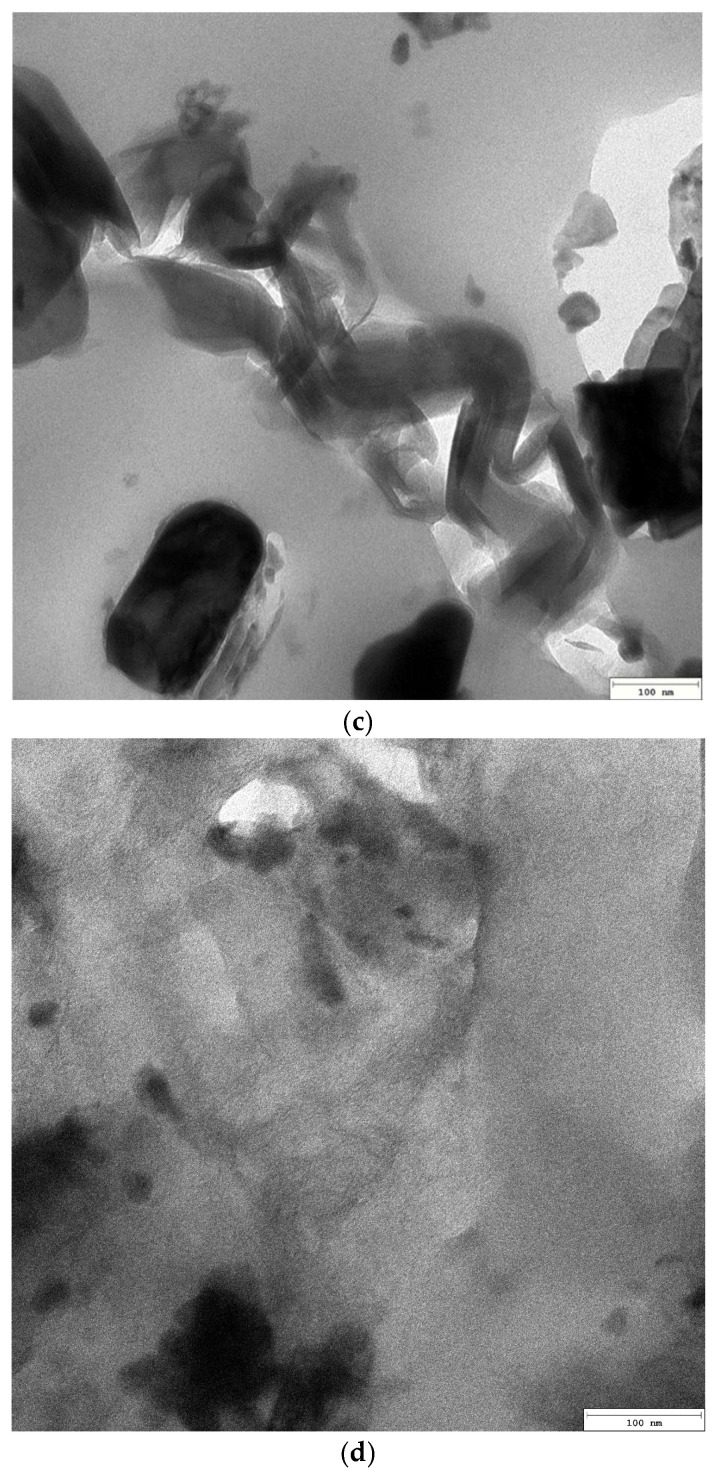
TEM images: (**a**) the original SWCNTs, (**b**) EP/30Zn coating, (**c**) EP/10Zn/0.5CN/I coating, (**d**) EP/10Zn/0.5CN/II coating.

A TEM image of the original single-walled carbon nanotubes is shown in Figure 1a. Figure 1b–d showed the surface morphology of coatings without SWCNTs and with 0.5 wt% SWCNTs dispersed by methods I and II, respectively.

Figure 1a showed a single-walled graphene nanotube concentrate. Long nanotubes were gathered in clusters like tangled fibers. Figure 1b clearly showed that the coating surface contains a significant amount of zinc particles. However, epoxy resin did not completely encapsulate all zinc particles. Figure 1c demonstrated that the introduction of carbon nanotubes (SWCNTs) dispersed in a “first” manner (EP/10Zn/0.5CN/I) resulted in their incorporation as tangled fiber clusters. However, as illustrated in Figure 1d, dispersing the nanotubes in a “second” way (EP/10Zn/0.5CN/II) effectively dispersed the original fiber clusters and allowed them to be incorporated into the polymer structure as a homogeneous network. Carbon fibers (visible as single threads) can effectively connect zinc particles in the matrix.

### 3.3. Electrochemical Measurements

**Figure 2:**
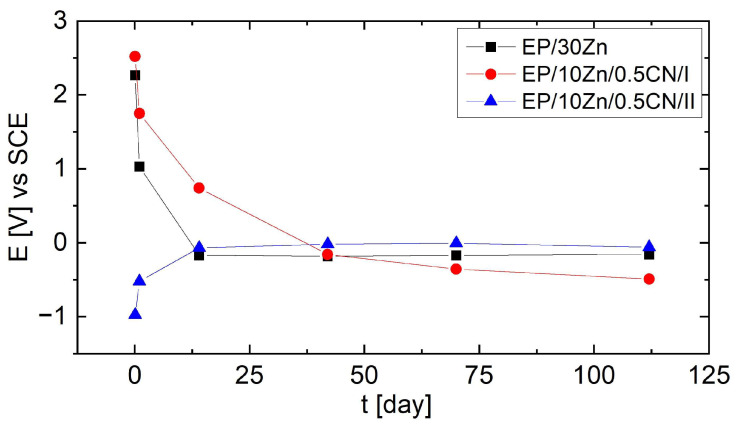
OCP evolution for coatings immersed in 3.5 wt% NaCl solution during 112 days.

The results of open circuit potential measurements conducted for 112 days are presented in Figure 2.

Based on the recorded potential values, it could be concluded that only the EP/10Zn/0.5CN/II coating showed a potential below −0.78 V vs. SCE in the initial stage of immersion (2 h), demonstrating the fully cathodic action of the coating towards the steel substrate. After 24 h, the OCP value for this coating increased to approximately −0.52 V, and after 14 days it stabilized slightly below −0.1 V. In the case of the EP/30Zn and EP/10Zn/0.5CN/I coatings, the recorded initial potential (OCP) was much higher (above 2 V) and decreased to −0.16 V and −0.49 V after 112 days, respectively. The changes for EP/10Zn/0.5CN/I occurred more gradually than for EP/30Zn.

**Figure 3:**
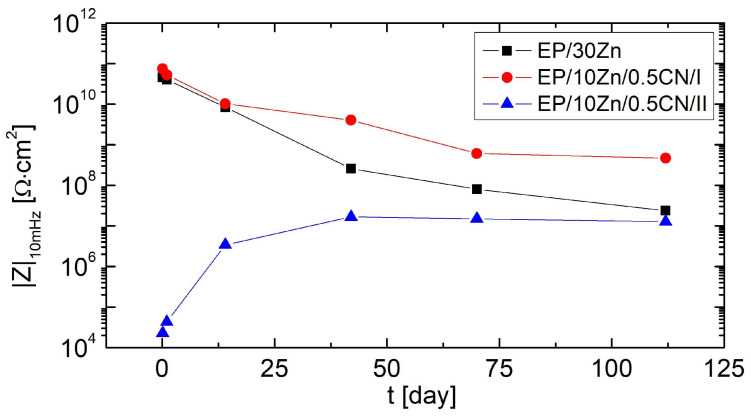
Evolution of impedance magnitude at 10 mHz for coatings during 112 days immersion in 3.5 wt% NaCl solution.

During 112 days of immersion in the corrosive solution, non-destructive electrochemical impedance measurements were performed successively. The impedance modulus values at the lowest frequencies (|Z|_10mHz_) are presented in Figure 3.

The EP/30Zn and EP/10Zn/0.5CN/I coatings exhibited very high impedance values (over 10 GΩ·cm^2^) from the beginning of conditioning. Approximately 14 days after immersion, the |Z|_10mHz_ values began to decrease significantly, reaching 24 MΩ·cm^2^ and 465 MΩ·cm^2^ after 112 days, respectively. This behavior was characteristic of barrier organic coatings, which became penetrated by the electrolyte over time, reducing their barrier properties. By the time the measurements were completed, the EP/10Zn/0.5CN/I coating exhibited impedance values nearly 20 times higher at low frequencies, which confirmed its more effective barrier action. In contrast, the EP/10Zn/0.5CN/II coating initially exhibited low impedance values (below 30 kΩ·cm^2^). In this case, with increasing conditioning time, the coating impedance increased rapidly, reaching 3.4 MΩ·cm^2^ after 14 days. After this time, the rate of change slowed. The maximum impedance value was obtained after 42 days of immersion in the corrosive medium (|Z|_10mHz_ = 17 MΩ·cm^2^). After this time, the impedance value of the EP/10Zn/0.5CN/I coating decreased slightly and reached 13 MΩ·cm^2^ after 112 days of measurement. This pattern of changes suggested that cathodic protection occurred initially, followed by barrier protection (the corrosion products formed seal the organic coating).

**Figure 4:**
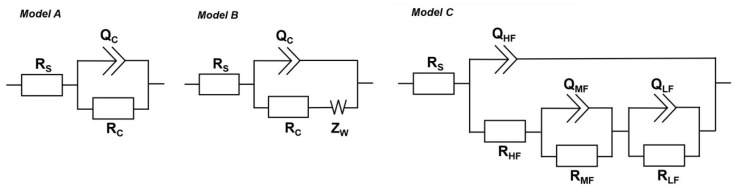
Equivalent electrical circuit diagrams for EIS fitting for: EP/30Zn and EP/10Zn/0.5CN/I coatings—model A (in the initial stage) and model B (at a later stage); EP/10Zn/0.5CN/II—model C.

To explain the anticorrosion mechanisms of the tested coatings, three equivalent electrical circuit (EEC) models were used, shown in Figure 4. These models were used to fit the EIS results.

Model A was selected to describe the corrosion processes that occurred in EP/30Zn and EP/10Zn/0.5CN/I coatings in the initial period after immersion in the corrosive medium. This was a typical equivalent electrical circuit model used to describe the impedance of organic coatings acting as a barrier. It consisted of the electrolyte resistance (*R*_S_), organic coating resistance (*R*_C_), and constant phase elements that represent the coating capacitance (*Q*_C_). In case of extending the conditioning time in the corrosive solution, it was necessary to introduce the Warburg impedance (*Z*_W_) to account for the effect of diffusion. At this stage, model B was used in simulation. In the case of the EP/30Zn coating, model B was applied beginning on the 14th day after immersion, while for the EP/10Zn/0.5CN/I coating, beginning on the 70th day. For the EP/10Zn/0.5CN/II coating, the spectra were analyzed and fitted using model C. It described impedance spectra with three time constants, which were present at different frequencies. The resistance (*R*_HF_) and constant phase element (*Q*_HF_) were used for processes at the highest frequencies (100 kHz to 1 kHz); the resistance (*R*_MF_) and constant phase element (*Q*_MF_) were used for processes at medium frequencies (1 kHz to 10 Hz); and the resistance (*R*_LF_) and constant phase element (*Q*_LF_) were used for processes at the lowest frequencies (10 Hz to 10 mHz).

**Figure 5:**
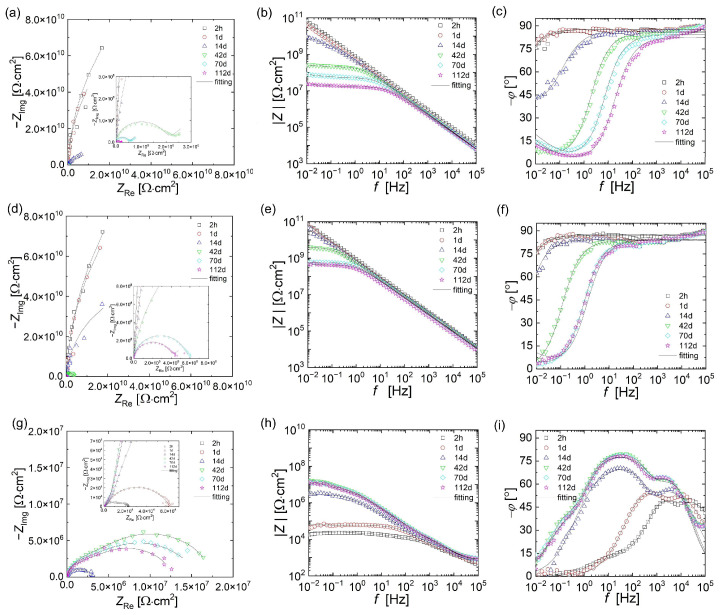
Nyquist and Bode plots of coatings during 112 days immersion in 3.5 wt% NaCl solution: (**a**–**c**) EP/30Zn, (**d**–**f**) EP/10Zn/0.5CN/I, (**g**–**i**) EP/10Zn/0.5CN/II.

Figure 5 shows the experimental impedance spectra of the tested coatings (points) along with the fitting results (lines). The values of individual parameters are summarized in Table 3 (EP/30Zn and EP/10Zn/0.5CN/I coatings) and Table 4 (EP/10Zn/0.5CN/II coating).

A good fit of the simulation results to the experimental measurement results and low *Chi*^2^ values indicated the correct selection of the equivalent electrical circuit models.

The initial resistance (*R*_C_) of the EP/30Zn and EP/10Zn/0.5CN/I coatings was very high (on the order of 100 GΩ·cm^2^), indicating that the coatings acted as barrier layers and charge transfer processes did not occur on the steel substrate. In the case of the EP/30Zn coating, this value decreased (by two orders of magnitude) after 14 days. In addition to the capacitive loop, the Nyquist plot also revealed a diffusion component (at low frequencies) related to the limitations of mass transport through the protective layer. During this time, the coating capacity (*Q*_C_) increased due to electrolyte penetration. Based on this, it can be concluded that the coating containing 30% Zn (without nanotubes) did not exhibit cathodic protection at any time.

A similar resistance mechanism was observed for the EP/10Zn/0.5CN/I coating (with 10% Zn and 0.5% SWNCT dispersed in method I). In this case, the lower zinc content (10%) compared to the EP/30Zn coating and the addition of filler (19.5%) and SWCNTs (0.5%) in its place enhanced the coating’s barrier effect. The coating resistance decreased significantly only after 42 days. At the end of the test (112 days), the resistance values remained high (*R*_C_ = 397 MΩ·cm^2^), compared to the EP/30Zn coating (*R*_C_ = 16.6 MΩ·cm^2^). This suggested that the presence of filler and nanotubes decreased the porosity of the coating, making it more difficult for the electrolyte to penetrate. Dispersion of carbon nanotubes in the polymer matrix using method I did not activate zinc particles in the polymer coatings.

The appearance and nature of changes in the impedance spectra of the EP/10Zn/0.5CN/II coating revealed a completely different mechanism of action of the coating compared to the previous coatings. In the Bode plot obtained two hours after immersion in the solution (Figure 5i), three time constants were observed. At a frequency of approximately 25 kHz, the first phase angle maximum was observed, indicating the existence of the first time constant. This was likely related to processes occurring within the carbon nanotube network, such as their internal conductivity or interactions at their surface. Low resistance (*R*_HF_) and capacitance (*Q*_HF_) values indicated rapid charge flow. The conductive effect of the carbon nanotube network in the polymer matrix of the EP/10Zn/0.5CN/II coating was confirmed by the low surface resistivity value obtained (12.68 kΩ; see Table 2). The introduction of SWCNTs using Method II resulted in a change in the coating’s character from an insulator to a conductor. A second phase angle maximum appeared in the mid-frequency range (approximately 2.5 kHz), reflecting the second time constant. This process was likely related to the barrier properties of the main polymer matrix, including their coating capacitance (*Q*_MF_) and coating pore resistance (*R*_MF_). In the low-frequency range (100 Hz–100 mHz) of the phase angle graph, instead of a clear maximum, an inflection (at approximately 10 Hz) was observed, which represented the third time constant. The existence of the third time constant was more clearly visible in the Nyquist plot (Figure 5g), where a smaller capacitive loop can be seen at low frequency values. The lack of a clear maximum was related to its partial overlap with the second time constant. The *R*_LF_ and *Q*_LF_ parameters in this case corresponded to the charge transfer resistance and double layer capacitance processes associated with active zinc corrosion. The sacrificial effect of zinc was also confirmed by the OCP value (−0.97 V vs. SCE). At this stage, carbon nanotubes activated the zinc powder by forming conductive networks that provided cathodic protection.

After 1 day of immersion, the impedance spectrum of the EP/10Zn/0.5CN/II coating changed significantly. Only two-time constants were visible for high and mid frequencies (phase angle maxima at 10 kHz and 0.5 kHz). The resistance values of *R*_HF_ and *R*_MF_ increased from 4.5 to 8.0 kΩ·cm^2^ and 12.1 to 51.4 kΩ·cm^2^, respectively, compared to the first measurement. The significant increase in resistance at medium frequency over time was likely related to the formation of zinc corrosion products, which expand and block the gaps/pores in the polymer coating. In turn, the increase in the *R*_HF_ value can be associated with a decrease in the charge transfer efficiency in the SWCNT network, probably due to the increase in contact resistance between individual nanotubes due to local deposition of corrosion products originating from the zinc dopant and/or gradual structural degradation of the nanotube network. The time constant at low frequency values disappeared, which can be attributed to the end of active zinc dissolution (sacrificial action). This was also confirmed by the increase in the OCP value to −0.52 V vs. SCE (above −0.78 V, considered the limit value for cathodic protection of the steel substrate). This behavior suggested effective sealing of the polymer coating.

The impedance spectra recorded after 14 days still exhibited only two-time constants (at high and mid frequencies). The phase angle maxima had shifted to frequencies of 4 kHz and 25 Hz, respectively. The resistance of *R*_MF_ increased significantly to 3.11 MΩ·cm^2^_._ This behavior suggested effective sealing of the polymer coating, i.e., the mechanism of barrier protection.

In the impedance spectra taken after 42 days of the EP/10Zn/0.5CN/II coating in the corrosive solution, three time constants were again observed. In addition to the time constants at high and mid frequencies (which the phase angle maxima did not change), a new time constant appeared at very low frequencies (below 1 Hz; inflection point at 0.5 Hz). At this stage, the *R*_LF_ and *Q*_LF_ parameters can be associated with the charge transfer resistance and double layer capacitance at the interface between the coating and the steel substrate. After 42 days of immersion, the coating reached its maximum impedance value. The resistance values of *R*_HF_, *R*_MF_, and *R*_LF_ were 32.2 kΩ·cm^2^, 5.7 MΩ·cm^2^, and 11.9 MΩ·cm^2^, respectively.

Between days 42 and 112 of conditioning, the impedance of the coating decreased, suggesting its gradual degradation and loss of barrier protection.

### 3.4. Corrosion Protection Mechanism

**Figure 6:**
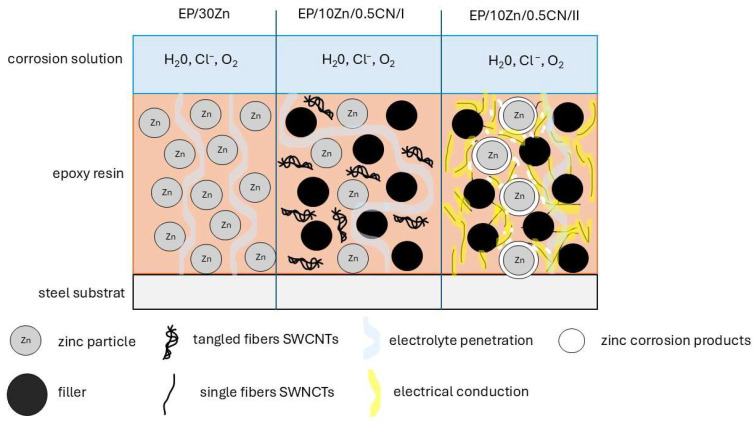
Schematic diagram of the coating corrosion protection mechanisms.

The proposed corrosion protection mechanism of the investigated coatings is presented in Figure 6.

#### 3.4.1. EP/30Zn Coating

A zinc content of 30% is insufficient to provide cathodic (sacrificial) protection; the coating functions primarily as a barrier. However, over time, it is gradually penetrated by the electrolyte, leading to a reduction in its impedance.

#### 3.4.2. EP/10Zn/0.5CN/I Coating

In this formulation, the zinc content was reduced from 30% to 10%, and replaced with 19.5% filler (barium sulfate) and 0.5% SWCNTs. The coating functions primarily as a barrier—nanotubes did not activate the zinc. The filler and the entangled SWCNTs act as obstacles to electrolyte penetration, forming a tortuous path that enhances barrier properties (maze mechanism).

#### 3.4.3. EP/10Zn/0.5CN/II Coating

In this case, the nanotubes were thoroughly dispersed within the polymer matrix, resulting in a dramatic increase in surface conductivity. Their contribution to the system’s conductivity was clearly visible in the impedance spectra at the highest frequencies. Within a few hours of immersion, the nanotube network activated the zinc particles. Zinc corrosion products likely formed not only directly on the zinc particles but potentially also within the conductive nanotube network, potentially diminishing its conductivity. These corrosion products (Zn(OH)_2_, ZnO, Zn_5_(CO_3_)_2_(OH)_6_), due to their larger volume compared to metallic zinc, could have contributed to sealing pores within the polymer coating.

Despite the rapid depletion of purely sacrificial (cathodic) protection, a mixed protection mechanism was likely to be in effect. The gradual oxidation of zinc continued to seal the coating structure. During the first 14 days, no characteristic time constant associated with processes on the steel substrate was observed in the impedance spectra, suggesting that corrosion processes were still taking place within the bulk of the coating. This indicated that the coating functioned through a barrier or a mixed mechanism. After 42 days, the appearance of a spectral region with high resistance at low frequencies suggested the onset of electrochemical processes on the steel substrate. In later stages, the system impedance gradually decreased, indicating the beginning of coating degradation.

The zinc dissolution process in a neutral solution can be expressed as follows [20]:

(2) $$O_{2}+{2H}_{2}O+{4e}^{-}\to {4OH}^{-}$$

(3) $$Zn-{2e}^{-}\to {Zn}^{2+}$$

(4) $$Zn+{2OH}^{-}\to {{Zn(OH)}_{2}+2e}^{-}$$

(5) $${6Zn(OH)}_{2}+{2CO}_{2}\to {{Zn}_{5}({CO}_{3})}_{2}{\left(OH\right)}_{6}+ZnO+{3H}_{2}O$$

### 3.5. Immersion Test

**Figure 7:**
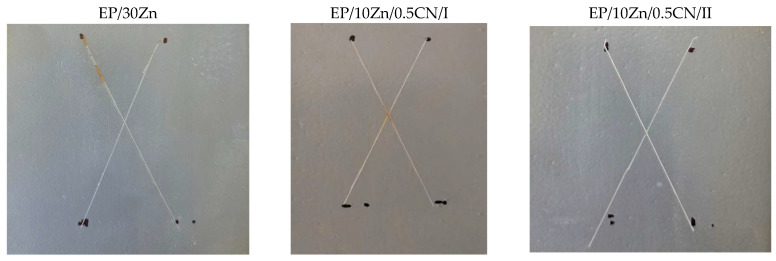
Photos of the coatings after the 240 h immersion test in 3.5% NaCl solution.

Figure 7 shows the appearance of the samples after 10 days in a 3.5% NaCl solution.

Based on the above images, red corrosion appeared in spots on samples EP/30Zn and EP/10Zn/0.5CN/I, while completely white corrosion products formed in the cuts on sample EP/10Zn/0.5CN/II. Based on this, it can be concluded that neither the 30% zinc content (in the EP/30Zn coating) nor the 10% zinc content, supported by 0.5% carbon nanotubes (SWCNTs) (EP/10Zn/0.5CN/I), provides fully effective cathodic protection of the steel substrate after the protective coating is damaged. However, this 10% zinc content, after nanotube dispersion using the “second” method (EP/10Zn/0.5CN/II), is sufficient for effective cathodic protection of the steel substrate, which manifests itself only in the formation of zinc corrosion products.

## 4. Conclusions

The use of SWCNTs had enabled the development of powdered epoxy primers with significantly lower zinc content (10 wt%) compared to conventional zinc-rich primers (i.e., 30–90 wt%), while maintaining—and even improving—anti-corrosion and mechanical properties.

The physical, mechanical, and protective properties of the coatings depended strongly on the method used for dispersing SWCNTs. The most effective dispersion approach involved initially dispersing the SWCNTs in molten epoxy resin, followed by cooling. The resulting SWCNT–resin mixture was then combined with all other raw materials, ground, and homogenized in a twin-screw co-rotating extruder. After extrusion, the material was cooled, ground, and sieved to obtain a low-zinc epoxy powder coating primer.

Reducing the Zn content from 30 wt% to 10 wt% with the simultaneous introduction of SWCNTs resulted in changes in the physicochemical properties of the coatings. Among other things, the current intensity in the powder cloud, roughness, water contact angle, and the amount of Zn released of seawater solution were reduced. The greatest changes occurred in surface resistivity and anti-corrosion properties. The introduction of nanotubes in Method I improved the coating’s barrier properties. The nanotubes in the agglomerates acted as a filler, hindering electrolyte penetration into the polymer coating. In the case of dispersion Method II, the nanotubes changed the coating’s character from barrier to conductive, ensuring cathodic and mixed activity with a low zinc content in the coating. In this case, an increase in impedance was observed within the first 42 days of immersion, which can be explained by the coating being sealed by zinc corrosion products. After this time, the coating’s impedance stabilized, which may result in improved anti-corrosion properties over time compared to other coatings (which provide barrier protection from the outset). From the point of view of using the coating as a low-zinc primer, Method II seems to be appropriate-reducing the zinc content while maintaining partial cathodic effect.

## Figures and Tables

**Table 1:** Qualitative/quantitative composition of the powder coatings.

Component/ Symbol of Coating	Epoxy Resin, wt%	Curing Agent, wt%	Barite wt%	Benzoin wt%	Byk 360 P wt%	Zinc Dust wt%	SWCNT wt%
EP/30Zn	65.07	3.43	-	0.5	1.0	30.0	-
EP/10Zn/0,5CN/I	65.07	3.43	19.5	0.5	1.0	10.0	0.5
EP/10Zn/0,5CN/II	65.07	3.43	19.5	0.5	1.0	10.0	0.5

**Table 2:** Specifications of physical and mechanical coatings properties.

Symbol of Coating		EP/30Zn	EP/10Zn/0.5CN/I	EP/10Zn/0.5CN/II
Current intensity in the powder cloud	µA	8.4 ± 0.3	5.4 ± 0.2	6.3 ± 0.2
Roughness PN-EN ISO 12085 [41]	*R*_a_, µm *R*_z_, µm	0.76 ± 0.02 3.92 ± 0.45	0.66 ± 0.02 3.50 ± 0.12	0.49 ± 0.03 2.66 ± 0.17
Gloss for the angle of 60 deg PN-EN ISO 2813 [42]	GU	36.2 ± 0.9	34.1 ± 0.8	49.0 ± 1.1
Thickness	µm	98.7 ± 0.1	100.1 ± 0.1	103.0 ± 0.1
Relative hardness PN-EN ISO 1522 [44]	-	0.59 ± 0.02	0.64 ± 0.02	0.69 ± 0.02
Adhesion to the steel PN-EN ISO 2409 [45]	0-best 5-worst	0	0	0
Water contact angle PN-EN 828 [46]	deg	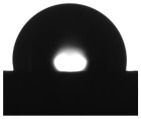 100.70 ± 1.10	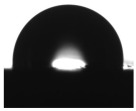 97.33 ± 1.05	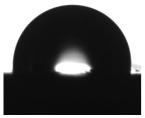 98.42 ± 1.21
Diiodomethan contact angle PN-EN 828 [46]	deg	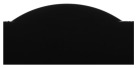 34.62 ± 0.71	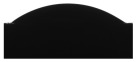 36.48 ± 0.96	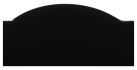 39.68 ± 0.92
Surface free energy	mN/m	46.63 ± 1.25	43.90 ± 1.20	42.29 ± 1.17
Surface resistivity EN ISO 3915 [47]	Ω	1.2 × 10^12^ ± 0.20 × 10^12^	1.3 × 10^9^ ± 0.20 × 10^9^	12.68 × 10^3^ ± 0.08 × 10^3^
Amount of Zn released in 100 cm^3^ of seawater solution	g/cm^2^	2.03 × 10^−4^ ± 0.03 × 10^−4^	1.33 × 10^−4^ ± 0.02 × 10^−4^	1.38 × 10^−4^ ± 0.03 × 10^−4^
Color PN-ISO 7724 [49]	-	L* = 47.57 ± 0.33 a* = −1.27 ± 0.04 b* = −3.10 ± 0.16 -	L* = 46.38 ± 0.36 a* = −1.16 ± 0.07 b* = −3.05 ± 0.15 ΔE* = 1.20 ± 0.09	L* = 44.64 ± 0.32 a* = −1.27 ± 0.04 b* = −3.43 ± 0.13 ΔE* = 3.48 ± 0.19

**Table 3:** EIS fitting parameters for EP/30Zn and EP/10Zn/0.5CN/I.

Coating	Time, day	*Q*_C_, S·s^n^/cm^2^	*n*_C_	*R*_C_, Ω·cm^2^	*Z*_W_, S·s^0.5^/cm^2^	*Chi* ^2^
EP/30Zn	0.08	2.16 × 10^−10^	0.972	3.25 × 10^11^	-	2.87 × 10^−3^
	1	3.39 × 10^−10^	0.974	2.39 × 10^11^	-	1.12 × 10^−3^
	14	4.07 × 10^−10^	0.961	3.62 × 10^9^	5.30 × 10^−10^	6.54 × 10^−3^
	42	4.73 × 10^−10^	0.951	1.99 × 10^8^	4.77 × 10^−8^	2.54 × 10^−3^
	70	5.75 × 10^−10^	0.937	5.02 × 10^7^	1.05 × 10^−7^	2.44 × 10^−3^
	112	7.62 × 10^−10^	0.918	1.66 × 10^7^	4.37 × 10^−7^	3.04 × 10^−3^
EP/10Zn/0.5CN/I	0.08	1.92 × 10^−10^	0.966	3.91 × 10^11^	-	8.22 × 10^−4^
	1	2.16 × 10^−10^	0.952	3.25 × 10^11^	-	2.87 × 10^−3^
	14	3.07 × 10^−10^	0.936	1.03 × 10^11^	-	6.03 × 10^−3^
	42	3.44 × 10^−10^	0.932	3.89 × 10^9^	-	2.02 × 10^−3^
	70	3.62 × 10^−10^	0.932	5.61 × 10^8^	6.57 × 10^−8^	1.70 × 10^−3^
	112	3.69 × 10^−10^	0.934	3.97 × 10^8^	3.36 × 10^−8^	1.81 × 10^−3^

**Table 4:** EIS fitting parameters for EP/10Zn/0.5CN/II.

Coating	Time, Day	*Q*_HF_, S·s^n^/cm^2^	*n*_HF_	*R*_HF_, Ω·cm^2^	*Q*_MF_, S·s^n^/cm^2^	*n*_MF_	*R*_MF_, Ω·cm^2^	*Q*_LF_, S·s^n^/cm^2^	*n*_LF_	*R*_LF_, Ω·cm^2^	*Chi* ^2^
EP/10Zn/0.5CN/II	0.08	1.87 × 10^7^	0.728	4.45 × 10^3^	8.10 × 10^−6^	0.571	1.21 × 10^4^	1.52 × 10^−8^	1.000	7.79 × 10^3^	1.70 × 10^−4^
	1	1.85 × 10^7^	0.735	1.17 × 10^4^	4.31 × 10^−8^	0.876	4.90 × 10^4^	-	-	-	1.61 × 10^−3^
	14	1.57 × 10^7^	0.743	3.49 × 10^4^	1.42 × 10^−8^	1.000	3.11 × 10^6^	-	-	-	5.27 × 10^−3^
	42	3.67 × 10^8^	0.874	3.22 × 10^4^	1.02 × 10^−8^	1.000	5.71 × 10^6^	1.53 × 10^−7^	0.837	1.19 × 10^7^	6.73 × 10^−4^
	70	3.98 × 10^8^	0.862	2.51 × 10^4^	1.24 × 10^−8^	1.000	4.72 × 10^6^	1.74 × 10^−7^	0.737	1.09 × 10^7^	5.30 × 10^−3^
	112	4.67 × 10^8^	0.861	3.93 × 10^4^	1.10 × 10^−8^	1.000	4.40 × 10^6^	2.20 × 10^−7^	0.785	8.38 × 10^6^	1.35 × 10^−3^

## Data Availability

The original contributions presented in this study are included in the article. Further inquiries can be directed to the corresponding authors.
